# Novel Extracellular PHB Depolymerase from *Streptomyces ascomycinicus*: PHB Copolymers Degradation in Acidic Conditions

**DOI:** 10.1371/journal.pone.0071699

**Published:** 2013-08-12

**Authors:** Javier García-Hidalgo, Daniel Hormigo, Miguel Arroyo, Isabel de la Mata

**Affiliations:** Department of Biochemistry and Molecular Biology I. Faculty of Biology, Complutense University of Madrid, Madrid, Spain; University of Padova, Medical School, Italy

## Abstract

The ascomycin-producer strain *Streptomyces ascomycinicus* has been proven to be an extracellular poly(*R*)-3-hydroxybutyrate (PHB) degrader. The *fkbU* gene, encoding a PHB depolymerase (PhaZ*_Sa_*), has been cloned in *E. coli* and *Rhodococcus* sp. T104 strains for gene expression. Gram-positive host *Rhodococcus* sp. T104 was able to produce and secrete to the extracellular medium an active protein form. PhaZ*_Sa_* was purified by two hydrophobic interaction chromatographic steps, and afterwards was biochemically as well as structurally characterized. The enzyme was found to be a monomer with a molecular mass of 48.4 kDa, and displayed highest activity at 45°C and pH 6, thus being the first PHB depolymerase from a gram-positive bacterium presenting an acidic pH optimum. The PHB depolymerase activity of PhaZ*_Sa_* was increased in the presence of divalent cations due to non-essential activation, and also in the presence of methyl-β-cyclodextrin and PEG 3350. Protein structure was analyzed, revealing a globular shape with an alpha-beta hydrolase fold. The amino acids comprising the catalytic triad, Ser^131^-Asp^209^-His^269^, were identified by multiple sequence alignment, chemical modification of amino acids and site-directed mutagenesis. These structural results supported the proposal of a three-dimensional model for this depolymerase. PhaZ*_Sa_* was able to degrade PHB, but also demonstrated its ability to degrade films made of PHB, PHBV copolymers and a blend of PHB and starch (7∶3 proportion wt/wt). The features shown by PhaZ*_Sa_* make it an interesting candidate for industrial applications involving PHB degradation.

## Introduction

Polyhydroxyalkanoates (PHAs) are intracellular polymers accumulated by a wide range of bacteria and archaea as a carbon and energy source when environmental conditions are not optimal for cell growth. Among these biopolymers poly(*R*)-3-hydroxybutyrate (PHB) is the best known and most common polyhydroxyalkanoate.

PHB has attracted much interest from the industry in the last two decades, since it is a natural thermoplastic that can be produced from renewable sources by different microorganisms or plants; it is biocompatible, nontoxic, biodegradable and presents some interesting chemical and mechanical properties, therefore PHB represents an interesting alternative to petroleum-derived plastics, despite its considerably higher production costs. Another important application of PHAs is related to the monomeric composition of this family of biopolymers, since all PHAs are enantiomerically pure polymers (all the monomers present *R* configuration). Thus, the degradation products of the PHAs are (*R*)-3-hydroxyalkanoic acids, valuable synthons for the chemical and pharmaceutical industries.

In the producer cell cytoplasm PHAs are stored in complex subcellular structures included under the term carbonosomes [1], which comprise amorphous PHA coated with a phospholipidic monolayer and different proteins involved in PHB production (polymerases), stabilization (phasins) or mobilization (depolymerases). These PHB carbonosomes are also known as native PHB or nPHB granules. When these granules are released into the extracellular medium as a consequence of cell lysis, PHAs become denatured, acquiring a semi-crystalline structure, known as denatured PHB or dPHB.

Insoluble dPHB granules are degraded in virtually every natural environment by extracellular PHB depolymerases produced by a wide variety of microorganisms, mainly bacteria and fungi. PHB depolymerases are specific for dPHB or nPHB, thus they are not able to degrade both types of PHB granules, except for the PHB depolymerase from *Bacillus megaterium*, classified as an intracellular nPHB depolymerase associated to PHB carbonosomes, but exhibiting dPHB depolymerase activity as well [2].

Many of PHB degrading microorganisms present in soil ecosystems are classified into the genus *Streptomyces* [3], which has been one of the most biotechnologically relevant genus during the last decades due to its metabolic versatility and the production of over 7000 natural antibiotics and other important bioactive compounds [4].

*fkbU* gene from *S. ascomycinicus* was described as a part of the FK520 gene cluster [5], responsible for the biosynthesis of ascomycin, a macrolide with immunosuppressive and antifungal activities. *fkbU* was proposed to encode a PHB depolymerase, but no experimental evidence regarding this enzyme was previously provided.

In this work, we demonstrate that *S. ascomycinicus* is able to degrade PHB and the identity of *fkbU* gene has been confirmed. In this sense, *fkbU* was cloned in the heterologous host *Rhodococcus* sp. T104, and its gene product, hereafter PhaZ*_Sa_*, was proven to be an extracellular dPHB depolymerase, which has been expressed in an active and extracellular form. Furthermore this depolymerase was purified as well as biochemically and structurally characterized, and a three-dimensional model was proposed for the tertiary structure of PhaZ*_Sa_*. Additionally, PhaZ*_Sa_* was used to perform film degradation tests employing pure PHB and PHB copolymers containing different monomeric contents of 3-hydroxyvalerate, as well as a blend of PHB and starch that has been reported to confer improved mechanical properties compared to PHB homopolymer, and also would allow industry to reduce the production costs of this kind of biodegradable plastics [6].

## Materials and Methods

### Chemicals

Cell culture medium reagents were from Difco (Becton Dickinson). All chemical reagents and polymers were purchased from Sigma-Aldrich.

### Bacterial Strains, Media, and Growth Conditions

All strains used in this study are summarized in table 1. *Streptomyces ascomycinicus* sp. nov. DSMZ 40822 [8], (formerly known as *S. hygroscopicus* subsp. *hygroscopicus* or *S. hygroscopicus* subsp. *ascomyceticus* ATCC 14891), described as a putative extracellular PHB depolymerase producer, was used as chromosomal DNA source. *Streptomyces exfoliatus* DSMZ 41693 [9], [10], was used as positive control and *Streptomyces coelicolor* CECT 3243 as negative control for degradation of PHB. *Escherichia coli* DH5α was used as host for subcloning experiments, *E. coli* BL21(DE3) and wild type strain *Rhodococcus* sp. T104 KACC 21099 were used as hosts for gene expression [9], [11]. *E. coli* cells were grown in Luria–Bertani (LB) medium at 37°C, supplemented, when necessary, with 1 mM IPTG to induce overexpression of the cloned genes. For DNA purification, *S. ascomycinicus* cells were sporulated in solid SFM (Soya Flour Mannitol) medium and cultured aerobically under submerged conditions in S-YEME liquid medium (yeast extract/malt extract/0.5% glycine to allow dispersed growth) at 30°C and 250 rpm [12]. For PHB depolymerase extracellular activity detection, *S. ascomycinicus* spores previously collected and washed with 0.9% (wt/vol) NaCl were grown in solid basal mineral medium [13] supplemented with 1 mg/ml PHB as sole carbon source; plates were incubated for 120 hours at 30°C. *Rhodococcus* sp. T104 cells were grown in 2×YT (yeast extract/bactotriptone/NaCl) medium supplemented with glucose (5 g/l) [12].

**Table 1:** Bacterial strains, plasmids and constructs used in this study.

Strain or plasmid	Relevant genotype or description	Reference
**Strains**		
*Escherichia coli* DH5α	F^−^ φ80d*lac*ZΔM15 *end*A1 *rec*A1 *hsd*R17 (r_k_^−^m_K_^+^) *sup*E44 *thi*-1 *gyr*A96 *rel*A1 Δ(*lac*ZYA-*arg*F)U169 λ^−^	[7]
*Escherichia coli* BL21(DE3)	F^–^ *omp*T *gal dcm lon hsd*S_B_(r_B_^−^ m_B_^−^) λ(DE3 [*lac*I *lac*UV5-T7 gene 1 *ind*1 *sam*7 *nin*5])	Invitrogen
*Streptomyces ascomycinicus*DSMZ 40822	PHB depolymerase producer	[5], [8]
*Streptomyces exfoliatus*DSMZ 41693	PHB and PHO depolymerase producer, used as positive control	[10]
*Rhodococcus* sp. T104KACC 21099	Wild strain, suitable for gene cloning/expression with pNV19 vector	[11]
**Plasmids**		
pET28	Cloning/expression vector for *E. coli* strains. 5.4 kb	Novagen
pHPET	pET28 derivative containing *fkb*U gene	This work
pEM4	Shuttle vector *E. coli*/*Streptomyces*. Ap^R^ Tsr^R^ p*erm*E* pUCo*ri* pWHM4ori 7.9 kb	[14]
pHPEM	pEM4 derivative containing *fkb*U gene	This work
pNV19	Shuttle vector *E. coli*/*Rhodococcus*. Km*^R^* pAL5000ori *lac*Z CoE1ori. 4.4 kb	[9], [15]
pHPNV	pNV19 derivative containing *fkb*U gene under control of *erm*E* promoter	This work
pHPNV S131A/S131C/D209N/H269E/H269Q	pHPNV derivatives with mutated codons encoding catalytic amino acids	This work

### Plasmids, DNA Manipulation and Sequencing

All plasmids used in this study are summarized in table 1. pET28a(+) (Km^R^, T7 promoter, *lacI*) (Novagen) was used for gene expression in *E. coli* BL21(DE3). Bifunctional pEM4 (Ap^R^, Tsr^R^, permE*) [14] and pNV19 (Km^R^, *lacZ*) [15] plasmids were used to obtain the recombinant vectors for gene expression in *Rhodococcus* sp. T104. Chromosomal DNA from *S. ascomycinicus* DSMZ 40822 was purified according to the method described elsewhere [12]. Plasmid DNA preparations, restriction endonuclease digestions, ligations, and other DNA manipulations were carried out according to standard procedures for *E. coli* [16] and *Streptomyces* [12]. DNA sequences were determined by the dideoxy-chain-termination method [17] with an automated sequencer, DNA Analyzer 3730 (Applied Biosystems).

### Construction of Strains Expressing the *fkbU* Gene

The putative PHB depolymerase encoding DNA sequence *fkbU*, (GenBank accession number: AF235504.1) was amplified by PCR using chromosomal DNA from *S. ascomycinicus* DSMZ 40822 as template. The PCR primers were designed according to the DNA sequence of *fkbU* [5]. Restriction sites *Nco*I, *Xba*I, and *Eco*RI were included in the primers to facilitate subcloning of PCR fragments. A *Streptomyces* RBS consensus sequence (GGAGG) was included in HPEM primer. PCR amplifications were performed in a Mastercycler Personal thermocycler (Eppendorf), employing *Pfu* DNA polymerase (Promega). The PCR products were purified by High Pure PCR Product Purification Kit (Roche), digested with endonucleases *Nco*I or *Xba*I and *Eco*RI, and cloned into the *Nco*I–*Eco*RI site of pET28 vector, resulting to recombinant plasmid pHPET or into the *Xba*I–*Eco*RI site of pEM4 vector, resulting to recombinant plasmid pHPEM. Recombinant plasmid pHPET was used to transform competent *E. coli* BL21(DE3) cells by heat shock. Recombinant pHPEM plasmid was digested with *Hin*dIII and *Eco*RI in order to obtain the fragments containing the ORF along with the strong ermE* promoter of the erythromycin resistance gene from *Saccharopolyspora erythraea* [18], [19] and transferred to the pNV19 vector, obtaining the recombinant plasmid pHPNV which was used to transform electrocompetent *Rhodococcus* sp. T104 cells, as previously described [9]. All resulting recombinant plasmids were purified by the High Pure Plasmid Isolation Kit (Roche) and sequenced to confirm the absence of mutations and the correct orientation.

### Production and Purification of PhaZ*_Sa_*

Recombinant *Rhodoccocus* sp. T104 (pHPNV) cells were cultured aerobically under submerged conditions in 1 liter 2×YTG with 100 µg/ml kanamycin at 30°C for 72 h at 250 rpm orbital shaking. Ammonium sulfate was added to the cell-free culture broth at a final concentration of 0.5 M. Then, the solution was centrifuged at 10,000×*g* for 10 minutes and loaded onto a 100 ml Octyl FF sepharose column (GE Healthcare) equilibrated with 20 mM potassium phosphate buffer pH 7.0, 0.5 M ammonium sulfate (Buffer A) using a BioLogic LP chromatographic system (Bio-Rad). The column was extensively washed with 100 ml of the same buffer and the retained proteins were eluted with a linear decreasing gradient of 0.5 to 0 M ammonium sulfate in buffer A. The protein fractions containing PhaZ*_Sa_* were pooled and ammonium sulfate was added until final concentration of 0.5 M. After centrifugation at 10,000×*g* for 10 minutes, the supernatant was loaded onto a HiTrap Phenyl HP sepharose cartridge (GE Healthcare) (1 ml bed volume) equilibrated with buffer A. The cartridge was washed with 7 ml of the same buffer and a decreasing linear gradient from 0.5 to 0 M ammonium sulfate allowed elution of the bound proteins (purification table is shown in table S2). Purity of the fractions showing PHB depolymerase activity was analyzed by SDS-PAGE [20]. The amount of protein in the enzyme solutions was routinely determined by the Coomassie Blue method [21].

### PHB Depolymerase Assays

Extracellular PHB depolymerase activity was measured by spot test or using the turbidimetric method, as described [9], [22] but using 50 mM MES buffer pH 6. One unit (U) of PHB depolymerase activity is the amount of enzyme needed to catalyze the decrease of 0.01 absorbance units (at 600 nm) per minute in the assay conditions described.

Effects of pH on PhaZ*_Sa_* stability were assessed by incubating 2 µg of pure PhaZ*_Sa_* for 45 minutes at 40°C and pH values from 4 to 9 in 20 mM phosphate/citrate/borate buffer with constant ionic strength of 120 mM, adjusted by addition of different amounts of NaCl.

Effects of temperature on PhaZ*_Sa_* stability were assessed by incubating 2 µg of pure PhaZ*_Sa_* for 45 minutes at temperatures ranging from 25 to 70°C in water bath with gentle shaking.

After the incubations, aliquots of enzyme were drawn at different times and placed on ice bath for five minutes. Remaining activity was immediately measured by the standard turbidimetric method. All assays were performed in triplicate.

PHB depolymerase activity of pure PhaZ*_Sa_* aliquots dialyzed against 50 mM MES buffer pH 6 was measured in the presence of different concentrations of divalent (MgCl_2_, CaCl_2_ and MnCl_2_) and monovalent (NaCl and KCl) cation chlorides by the standard turbidimetric assay. PhaZ*_Sa_* activity was also assessed by the standard turbidimetric assay in the presence of several concentrations of EDTA (with 2 mM MgCl_2_), methyl-β-cyclodextrin, polyethylenglycol 3350, reducing agents (DTT and 2-mercaptoethanol), corn starch and detergents (SDS, Tween 20 and Triton X-100), as well as in presence of twelve different organic solvents with 10% (vol/vol) concentration.

The apparent *K_m_* and *V_max_* values of PhaZ*_Sa_* for PHB hydrolysis were calculated by non-linear hyperbolic regression, using the starting values obtained by linear regression fitting of a Hanes-Woolf plot, [23], [24] with the Hyper32 software (freely available at http://homepage.ntlworld.com/john.easterby/hyper32.html). These parameters were calculated using the turbidimetric activity assay with PHB, the natural substrate of PhaZ*_Sa_*, and considering a PHB weight average molecular mass (M_W_) of 437 kDa, provided by the manufacturer.

The release of (*R*)-3-hydroxybutyrate by PhaZ*_Sa_* was measured using a spectrophotometric activity assay employing the β-hydroxybutyrate dehydrogenase from *Pseudomonas lemoignei* (Sigma) [22], [25]. Production of NADH as a result of (*R*)-3-hydroxybutyrate oxidation was measured at 340 nm after incubation of 100 µl samples for 30 minutes at 37°C with 9.5 mU of β-hydroxybutyrate dehydrogenase and 1 mM NAD^+^, in 75 mM Tris-HCl buffer pH 8, in a total volume of 500 µl. Reaction was stopped on ice and absorbance at 340 nm was immediately measured. Concentration of (*R*)-3-hydroxybutyrate was calculated by interpolating the absorbance values in a standard curve.

### Degradation of PHB and PHBV films by PhaZ*_Sa_*

Thin solvent-cast films of pure PHB, PHBV with 5 or 12% (wt) 3-hydroxyvalerate and PHB-starch 7∶3 (wt) proportion were prepared by dissolving 100 mg of the polymer or blend in 20 ml of chloroform with heating and vigorous stirring. The solutions were poured on glass Petri dishes and then chloroform was evaporated at room temperature overnight. The films were subsequently submerged in 20 ml of 150 mM MES buffer pH 6 with 5 mM MgCl_2_ and 4 mM MβCD. Finally 250 µl of enzyme solution containing 30 µg of PhaZ*_Sa_* or 20 mM potassium phosphate buffer pH 7 (in the case of controls) were added to each plate. Plates were incubated at 37°C for 40 hours. Aliquots and pictures were taken at different times in order to monitor the degradation of the films; (*R*)-3-hydroxybutyrate concentration in the aliquots was quantified by the β-hydroxybutyrate dehydrogenase assay.

### Chemical Modification of Recombinant PhaZ*_Sa_*

Modification by Phenylmethylsulfonyl fluoride (PMSF), diethylpyrocarbonate (DEPC), and 1-ethyl-3-(3-dimethylaminopropyl)carbodiimide (EDC) were performed incubating 5 µg of recombinant PhaZ*_Sa_* with the appropriate amount of the suitable group-specific reagent as described [9]. PMSF solution was prepared in DMSO, DEPC in ethanol, and EDC was dissolved in 30 mM MES buffer pH 5. The concentration of DMSO or ethanol in the enzymatic assay did not exceed 0.1% (vol/vol) and was found to have no noticeable effect on the stability or activity of the enzyme. The remaining enzyme activity was determined by the standard turbidimetric assay.

Sulfhydryl groups were quantified spectrophotometrically with DTNB (5,5′-dithiobis-(2-nitrobenzoic acid) by Ellman’s method [26]. All experiments were carried out in triplicate, and mean values are shown in the tables. Controls for enzyme activity were carried out in all experiments.

### Secondary Structure Elucidation

PhaZ*_Sa_* secondary structure was predicted using several bioinformatic tools or servers: PSIPRED (freely available at bioinf.cs.ucl.ac.uk/psipred) [27], Jpred 3 (freely available at www.compbio.dundee.ac.uk/www-jpred) [28] and PredictProtein, a collection of prediction tools available at www.predictprotein.org. Secondary structure content of PhaZ*_Sa_* was also experimentally obtained by circular dichroism spectrum deconvolution, using pure PhaZ*_Sa_* aliquots. Spectra were recorded with 102 µg/ml PhaZ*_Sa_* in 5 mM potassium phosphate buffer pH 7 at 25°C between 190 and 260 nm under thermostated conditions by using a JASCO J-715 spectropolarimeter. The CD readings were expressed as the mean residue molar ellipticity (degrees • cm^2^ • dmol^−1^), assuming a residue molecular mass of 104 Da according to the average amino acid molecular mass of PhaZ*_Sa_*. Secondary structure data was obtained from CD spectra deconvolution, using the CDNN V2.1 program [29]. Thermal unfolding of PhaZ*_Sa_* was analyzed by CD variation at 209 nm in 25–80°C range scanned at 20°C/h.

### Identification of the PhaZ*_Sa_* Reaction Products

After 18 hours reaction with 1 µg PhaZ*_Sa_* and 300 µg/ml PHB in 20 mM MES buffer pH 6 at 40°C and 300 rpm orbital shaking, reaction products were derivatized with bromophenacyl bromide (BPB), and subsequently detected and identified by HPLC-MS as described [9], [30].

### Site-directed Mutagenesis Studies

In order to ascertain the identity of the amino acids which constitute the catalytic triad of PhaZ*_Sa_*, the candidates chosen according to multiple sequence alignment were mutated using the Quikchange II XL site-directed mutagenesis kit (Stratagene). pHPNV plasmid was used as template for mutagenic PCR, using the primers listed in table S1. The resulting mutant constructions were sequenced to confirm the mutations and then transferred to *Rhodococcus* sp. T104 for protein expression. Serine 131 was exchanged for Alanine (mutant S131A) or Cystein (S131C); Aspartic acid 209 was exchanged for Asparagine (D209N), and Histidine 269 was exchanged for Glutamic acid (H269E) or Glutamine (H269Q).

### Analytical Ultracentrifugation Analysis

Aliquots of pure PhaZ*_Sa_* with three different concentrations (65, 129 and 259 µg/ml) in 25 mM potassium phosphate pH 7 with 100 mM NaCl were subjected to sedimentation velocity experiments with a Beckman Coulter XL-I analytical ultracentrifuge equipped with absorbance optics, at 48,000×*g* and 20°C, using an An-60Ti rotor and standard (12 mm optical path) double sector centerpieces of Epon-charcoal. Baseline offsets were measured afterwards at 200,000×*g*. The apparent sedimentation coefficient of distribution, c(*s*), and sedimentation coefficient *s* were calculated from the sedimentation velocity data using the SEDFIT software [31].

## Results

### Multiple Sequence Alignment of PhaZ*_Sa_* Amino Acid Sequence

**Figure 1:**
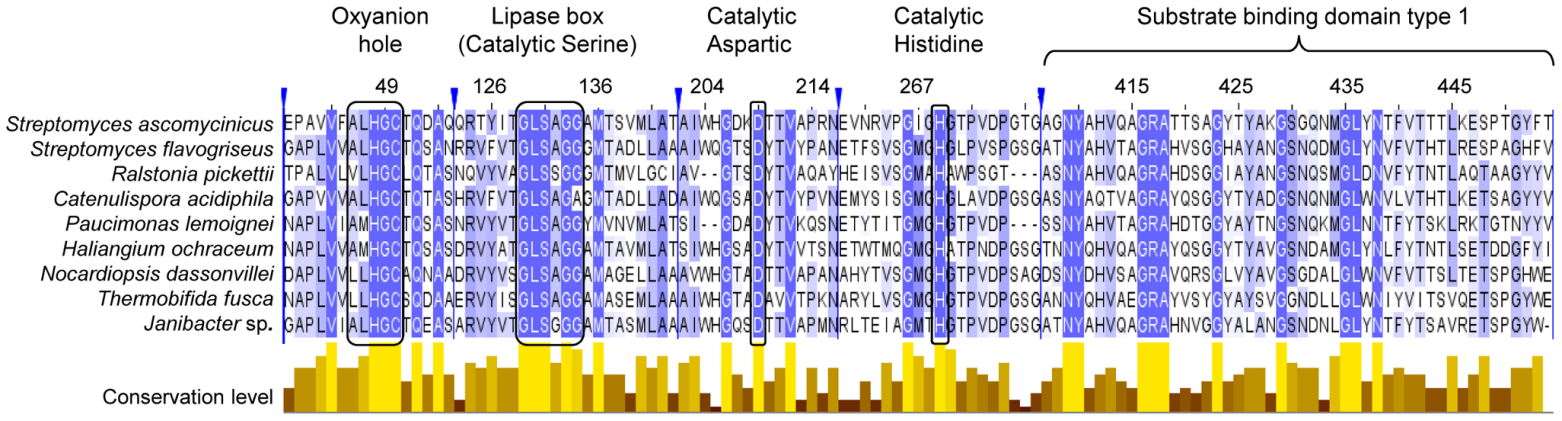
Multiple sequence alignment of PhaZ*_Sa_* with other PHB depolymerase sequences. Only regions with conserved amino acids are shown. All shown sequences present an identity below 58% among each other to avoid redundancy. Main catalytic amino acids are marked with black boxes. Conservation level of each position can be observed in the yellow bars below. Vertical blue lines represent gaps in the complete sequence.

Complete amino acid sequence of the putative PHB depolymerase PhaZ*_Sa_* (519 amino acids, accession number AAF86381.1) deduced from *fkbU* gene from *Streptomyces ascomycinicus* sp. nov. DSMZ 40822 [5], was analyzed and aligned with a selection of homologous PHB depolymerase sequences from different Gram-positive (*Catenulispora acidiphila, Streptomyces flavogriseus, Nocardiopsis dassonvillei, Thermobifida fusca* and *Janibacter* sp.) and Gram-negative (*Haliangium ochraceum, Paucimonas lemoignei* and *Ralstonia pickettii)* microorganisms (Figure 1). All chosen sequences present a maximum identity below 58% among each other, in order to minimize redundancy; sequence similarity/identity matrix was calculated using MatGAT v 2.01 [32]. Multiple sequence alignment was performed with T-Coffee software, freely available at www.tcoffee.org [33]. In this alignment some highly conserved regions can be found, including the putative oxyanion hole and lipase box (Gly-Leu-Ser-Ala-Gly). Putative catalytic amino acids (Ser^131^-Asp^209^-His^269^) are strictly conserved, as well as the putative histidine in the oxyanion hole (His^48^). PhaZ*_Sa_* shows the typical arrangement of extracellular dPHB depolymerases with secretion signal peptide, catalytic domain, fibronectin type III linking domain and a C-terminal substrate binding domain [34]. According to the PHA Depolymerase Engineering Database [35] available at http://www.ded.uni-stuttgart.de, PhaZ*_Sa_* belongs to the dPHAscl homologous family 11.

### Detection of PHB Depolymerase Activity in *S. ascomycinicus*

**Figure 2:**
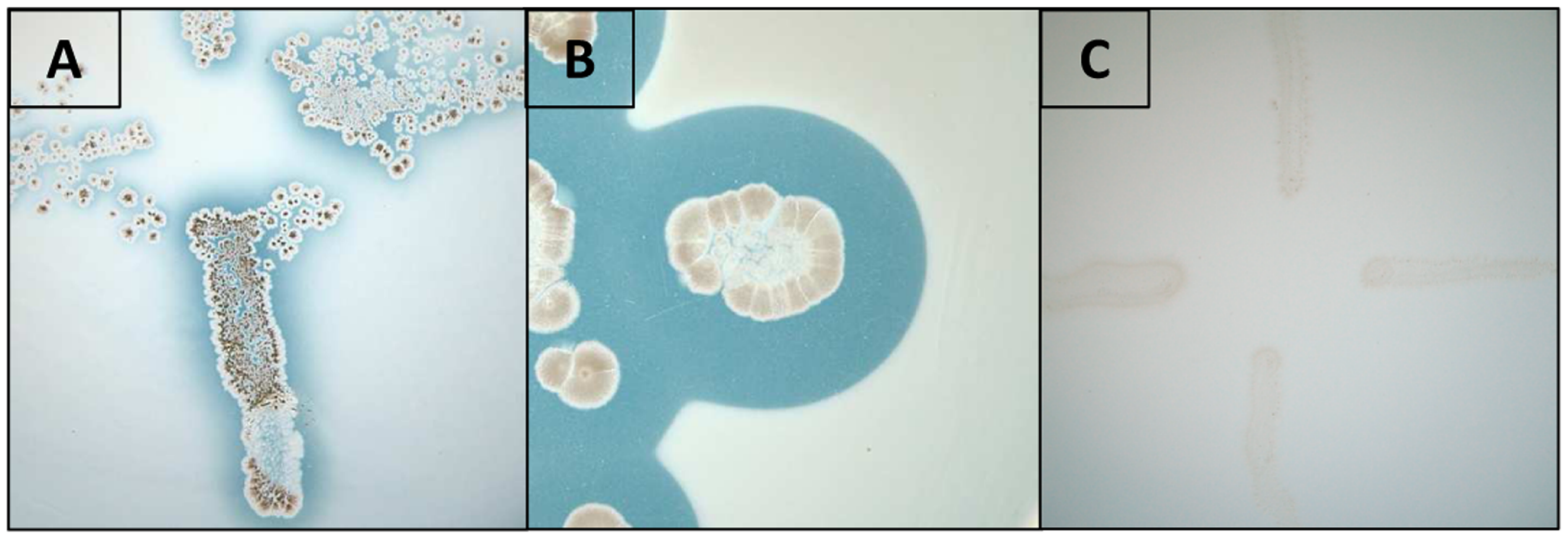
Detection of extracellular PHB depolymerase activity in *Streptomyces ascomycinicus*. Basal mineral medium plates supplemented with PHB were inoculated with fresh spores of *Streptomyces ascomycinicus* (A), *Streptomyces exfoliatus* (positive control) (B) or *Streptomyces coelicolor* (negative control) (C), and incubated for 120 hours at 30°C. The clear halos surrounding the microbial growth indicate the degradation of PHB.

The putative gene *fkbU* encoding PHB depolymerase from *S. ascomycinicus* was previously described [5], however no activity has been reported so far. In order to determine the extracellular PHB depolymerase activity of *S. ascomycinicus*, fresh spores were grown on solid basal mineral medium with PHB as sole carbon source, as described in materials and methods section. After 120 hours of incubation at 30°C a clear zone around the streak could be observed (Figure 2) demonstrating that *S. ascomycinicus* is able to degrade extracellular denatured PHB. This result shows the ability of this microorganism to produce an extracellular dPHB depolymerase.

### Construction of Strains Expressing the *fkbU* Gene and Detection of PhaZ*_Sa_*

The putative PHB depolymerase-coding gene *fkbU* was amplified by PCR and cloned into different vectors with the aim of expressing an active form of this enzyme. The pET28 derivative plasmid pHPET was used to clone and overexpress *fkbU* in *E. coli* BL21(DE3), but after inducing its expression with IPTG this recombinant strain was not able to produce an active form of PhaZ*_Sa_*, leading to the formation of insoluble inclusion bodies of PhaZ*_Sa_*.

In order to express *fkbU* gene in a homologous host, pHPEM plasmid was constructed, this pEM4 derivative plasmid contains the strong and constitutive ermE* promoter upstream the *fkbU* ORF, enabling the expression of this gene in gram positive hosts.

pHPEM was digested, and the *Hin*dIII-*Eco*RI fragment was cloned into pNV19, giving rise to the expression plasmid pHPNV, which was used to transform electrocompetent *Rhodococcus* sp. T104 cells. The recombinant *Rhodococcus* sp. T104 pHPNV cells were grown in 2×YTG medium and the PHB depolymerase activity of intracellular cell extract, insoluble cell debris and cell-free culture broth was assayed by the spot test method (Figure S1–B). Most of the activity was located in the fermentation broth, and a protein band of around 48 kDa corresponding to PhaZ*_Sa_* was detected by SDS-PAGE analysis of the fermentation broth, demonstrating that this enzyme was being secreted to the extracellular medium (Figure S1–A).

The N-terminal sequence of this extracellular protein obtained by Edman sequential degradation [36] was Ala-Ala-Gly-Leu-Ala-Lys-Pro-Gly-Leu-Thr-Lys-Ala-Asp-Leu-Thr-Glu-Val. Therefore, mature PhaZ*_Sa_* consists of 461 amino acids, with a theoretical mass of 48 kDa. It is noteworthy that the signal peptide is correctly recognized and cleaved by *Rhodococcus* sp. T104.

### Purification and Analysis of Recombinant PhaZ*_Sa_*

**Figure 3:**
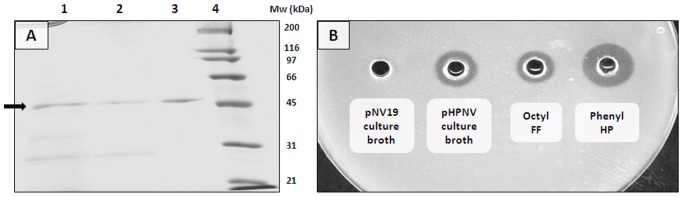
Analysis of recombinant PhaZ*_Sa_* purification steps. **A)** SDS-PAGE analysis: Lane 1: *Rhodococcus* T104 pHPNV culture broth; lane 2∶1 µg protein after octyl FF sepharose purification step; lane 3∶0.89 µg of purified protein after phenyl HP sepharose purification step; lane 4: Bio-Rad broad range molecular weight standards. Bands corresponding to PhaZ*_Sa_* are marked with an arrow. **B)** Spot test activity assay: *Rhodococcus* T104 pNV19 culture broth (negative control), *Rhodococcus* T104 pHPNV culture broth, pooled fractions after octyl FF sepharose purification step and pooled fractions after phenyl HP sepharose.

Recombinant PhaZ*_Sa_* produced by *Rhodococcus* sp. T104 pHPNV was purified by only two consecutive hydrophobic interaction chromatographic (HIC) steps (Figure 3) MALDI-TOF analysis of pure enzyme showed a main peak of 48.4 kDa which fits the theoretical value deduced from the sequence (48.0 kDa), and several minor peaks corresponding to different protein aggregation states (Figure S2), the peak of 24.2 kDa corresponds to the same form with double electric charge. In addition, aliquots of pure PhaZ*_Sa_* with three different concentrations (65, 129 and 269 µg/ml) were subjected to sedimentation velocity analysis to ascertain the expected monomeric nature of this enzyme. The experiments with all these preparations showed a single peak with an apparent molecular mass between 40 and 46 kDa, corresponding to the active monomeric form of PhaZ*_Sa_*, and also showed characteristic values of a globular shaped protein.

**Figure 4:**
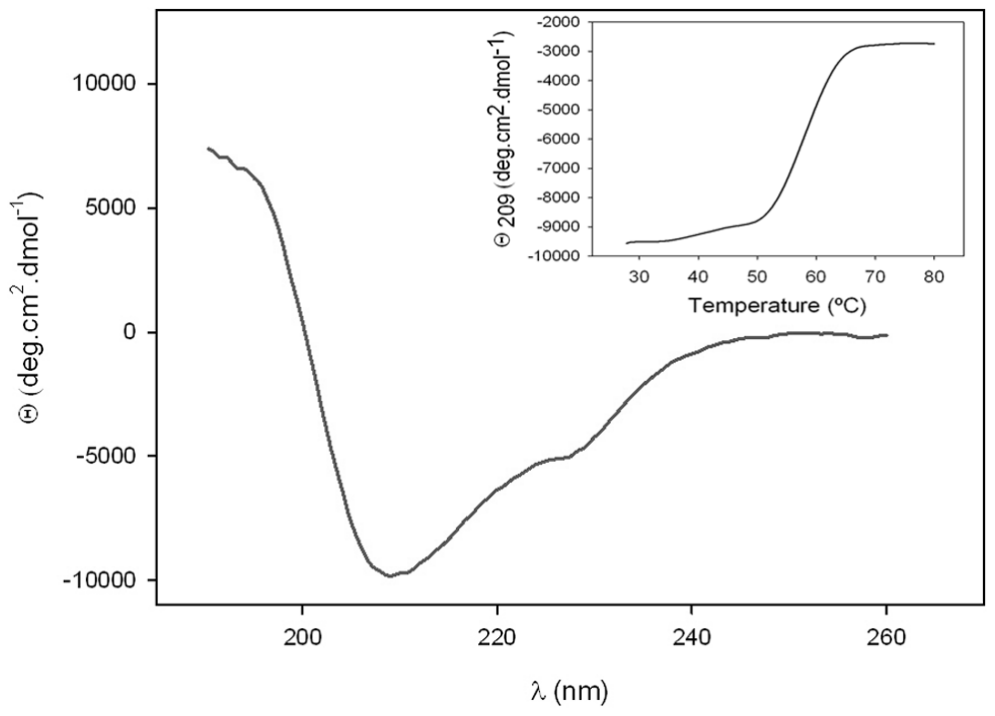
Far UV circular dichroism spectrum of pure recombinant PhaZ*_Sa_*. Inset: thermal unfolding of PhaZ*_Sa_*, studied by CD variation at 209.0 nm in 25–80°C range.

The secondary structure content of PhaZ*_Sa_* was experimentally calculated by CD spectrum deconvolution (Figure 4), and was also predicted according to the amino acid sequence with the online servers Jpred 3, PSIPRED and PredictProtein. Experimental and theoretical structural data were compared (Table 2). Experimentally obtained structural percentages differ slightly from the predicted values. However, all the programs employed for the prediction of secondary structure provide similar values, pointing to an alpha-beta structure.

**Table 2:** Structural composition percentages of PhaZ*_Sa_*.

Structure type	CD	PSIPRED	Jpred 3	PredictProtein
α-helix	16.8	12.4	11.5	12.8
β-sheet	32.2	27.1	29.9	26.5
Other	51.2	60.5	58.6	60.7

Comparison between the values deduced by CD spectrum deconvolution and those obtained by different secondary structure prediction servers.

Thermal denaturation of PhaZ*_Sa_* was also monitored by CD, measuring the change of ellipticity at 209 nm in a temperature range from 25 to 80°C (Figure 4 inset), showing a single melting temperature (*T*_m_) of 58.4°C.

### Biochemical Characterization of Recombinant PhaZ*_Sa_*

Recombinant PhaZ*_Sa_* was functionally characterized, showing highest activity at pH 6 and 45°C. It showed full stability at the entire range of pH values (4 to 9) and temperatures up to 50°C.

**Figure 5:**
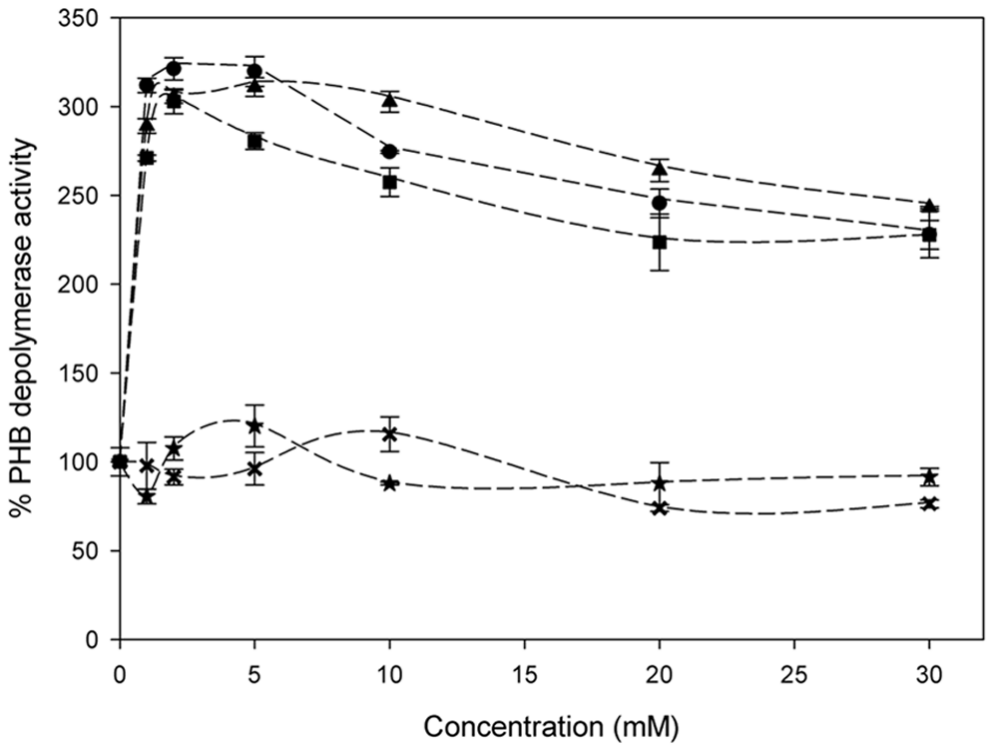
Activity of dialyzed PhaZ*_Sa_* in presence of different concentrations of Magnesium (▴), Calcium (•), Manganese (▪), Sodium (×) or Potassium (★) chlorides.

In addition, the effect of different cations on activity of dialyzed PhaZ*_Sa_* was also studied, (Figure 5), revealing a strong dependence on the presence of divalent cations such as magnesium, calcium or manganese. Monovalent cations did not exert evident effects on activity of PhaZ*_Sa_* at concentrations up to 30 mM, although the increase of ionic strength gradually inhibited the PHB depolymerase activity leading to its complete inactivation at 2.5 M NaCl. The activity of PhaZ*_Sa_* was also assayed in the presence of several organic solvents at 10% (vol/vol) (Table 3), as well as in the presence of different additives (Table 4). It is remarkable the slight effect of reducing agents on PhaZ*_Sa_* activity compared to other PHB depolymerases, [10], [37], [38], [39] and the notable inhibitory effect of EDTA, which was expected due to the high dependence of PhaZ*_Sa_* activity on divalent cations.

**Table 3:** PHB depolymerase activity of PhaZ*_Sa_* in the presence of 10% (vol/vol) of different organic solvents.

Solvent	Relative activity (%)
Control	100
Glycerol	97
Ethylene glycol	72
THF	49
Acetonitrile	30
Diethylene glycol	29
2-propanol	29
Triethylene glycol	24
Acetone	18
Ethanol	16
DMF	15
DMSO	14
Methanol	9

**Table 4:** Effect of different concentrations of several reagents on the PHB depolymerase activity of PhaZ*_Sa_*.

Reagent	Concentration	Relative activity (%)
Control	–	100
EDTA (+2 mM MgCl_2_)	1 mM	88
	10 mM	18
	20 mM	14
Methyl-β-cyclodextrin	1 mM	126
	5 mM	146
	10 mM	146
PEG 3350	1 mM	112
	5 mM	106
	10 mM	98
DTT	1 mM	90
	5 mM	44
	10 mM	27
2-Mercaptoethanol	1 mM	100
	5 mM	97
	10 mM	91
Corn starch	50 µg/ml	103
	200 µg/ml	99
	400 µg/ml	98
	1000 µg/ml	104
SDS	0.1%	Not detected
	1%	
Tween-20	0.1%	
	1%	
Triton X-100	0.1%	
	1%	

Furthermore, the kinetic parameters of recombinant PhaZ*_Sa_* for PHB hydrolysis were also determined. The apparent *K_m_* and *V_max_* values were 0.61±0.11 µM (269±48 µg/ml) and 9796.8±186.8 U/mg of enzyme, respectively.

**Figure 6:**
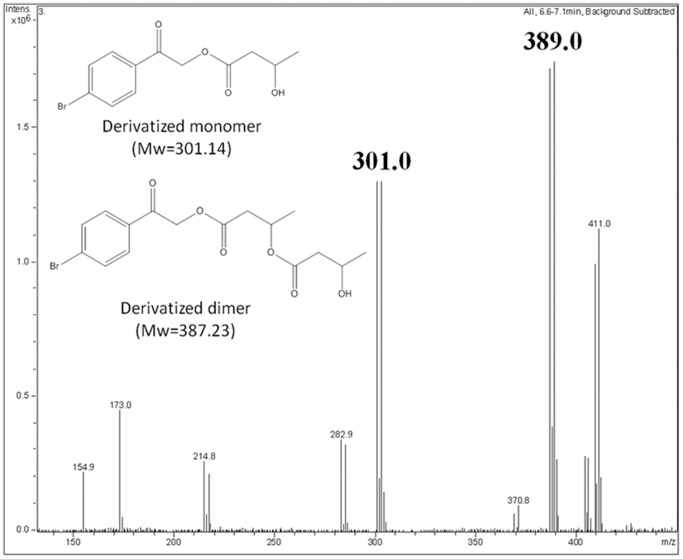
Identification of PhaZ*_Sa_* reaction products by HPLC–MS. The insets on the spectrum show the structure of the BPB derivatized compounds and their corresponding molecular weights. Double picks with similar intensity and a mass difference of 2 Da correspond to bromine-containing molecules, due to the isotopic abundance of this element (50.69% for Br^79^ and 49.31% for Br^81^).

Finally, the products released after PHB hydrolysis catalyzed by recombinant PhaZ*_Sa_* were derivatized with bromophenacyl bromide (BPB) and analyzed by HPLC-MS. As it can be observed in figure 6, derivatized monomers and dimers of (*R*)-3-hydroxybutyrate could be detected and identified in the reaction medium, suggesting that PhaZ*_Sa_* displays an exo-type hydrolysis mechanism.

### Degradation of PHB and PHBV Films by PhaZ*_Sa_*

**Figure 7:**
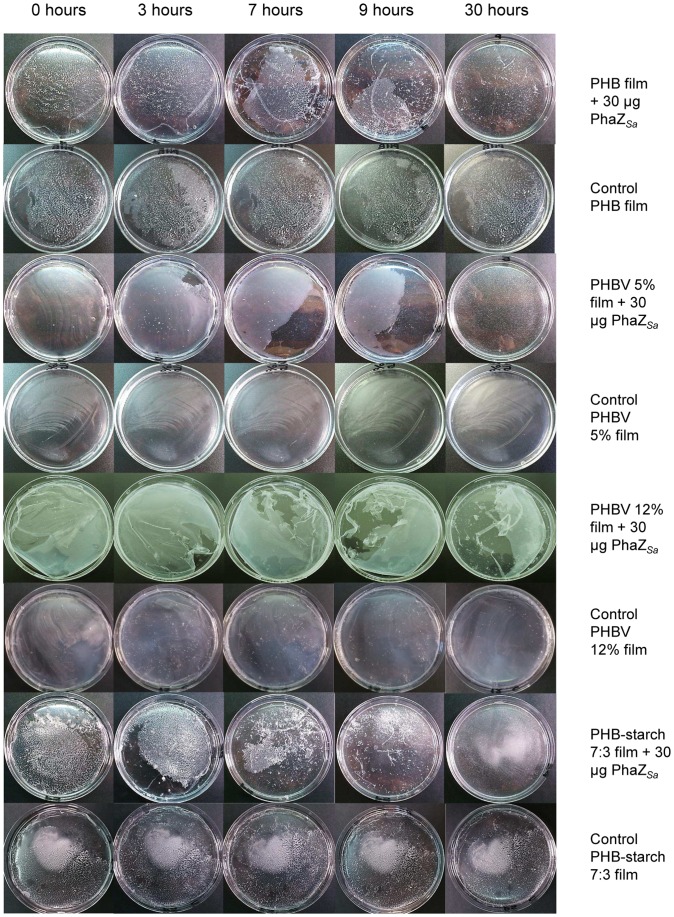
Degradation of different PHB films by PhaZ*_Sa_*. Solvent cast films of PHB, PHBV 5%, PHBV 12% and PHB-starch 7∶3 were incubated with 30 µg of PhaZ*_Sa_* at 37°C without shaking. Control films were incubated in the same conditions without enzyme. Pictures were taken at different times.

**Figure 8:**
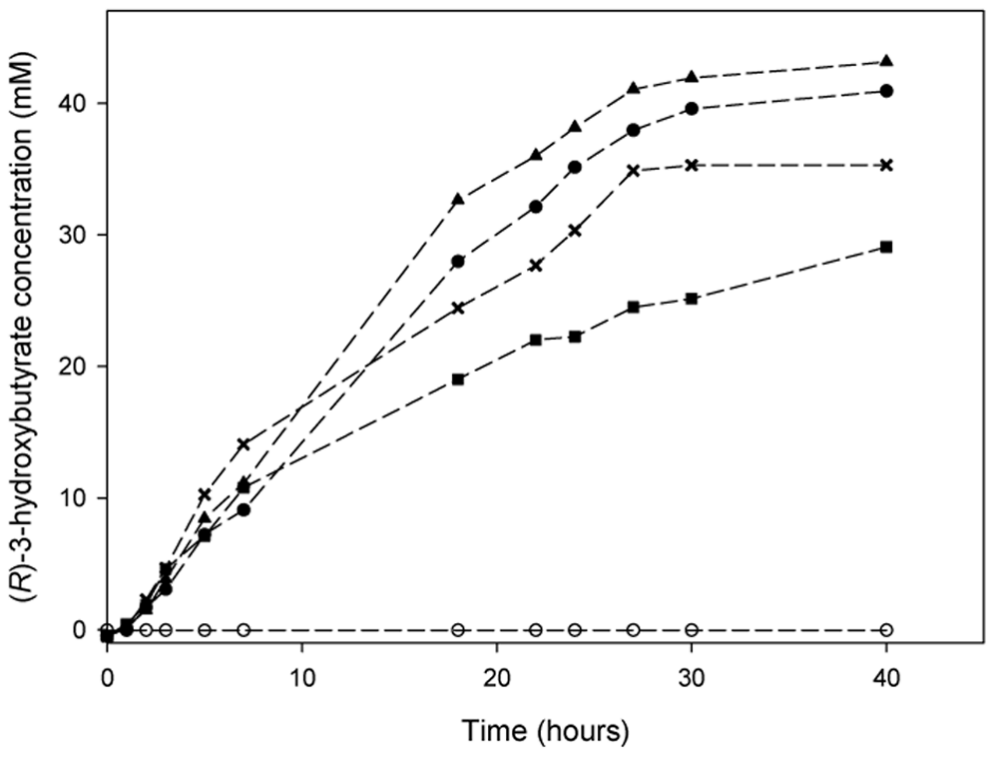
Release of (*R*)-3-hydroxybutyrate from different PHB films catalyzed by PhaZ*_Sa_*. Concentration of (*R*)-3-hydroxybutyrate, released from the films by PhaZ*_Sa_*, was monitored during the first 40 hours of reaction by the β-hydroxybutyrate dehydrogenase method. The films used were PHB (•), PHBV 5% (▴), PHBV 12% (▪), and PHB-starch 7∶3 proportion (**×**). All the controls without enzyme (O) presented undetectable concentrations of (*R*)-3-hydroxybutyrate.

The degradation by recombinant PhaZ*_Sa_* of films made of PHB, PHBV with 5 or 12% (wt) content of 3-hydroxyvalerate and PHB-starch 7∶3 (wt) proportion was assessed for 40 hours at 37°C and pH 6. Images of the films at different times are shown in figure 7. Release of (*R*)-3-hydroxybutyrate to the reaction medium was quantified by the β-hydroxybutyrate dehydrogenase method. As observed in Figure 8, PhaZ*_Sa_* was able to degrade all the films tested. However, depolymerization was slower in the case of PHBV 12% due to its high 3-hydroxyvalerate content. Films made of pure PHB, PHBV 5% and PHB-starch yielded a similar amount of product, and were completely disintegrated within the first 24 hours. Control films were incubated in the same conditions without enzyme to evaluate the possible spontaneous degradation of the polymers. In such cases, concentration of (*R*)-3-hydroxybutyrate was undetectable during the first 40 hours.

### Identification of Catalytic Amino Acids of PhaZ*_Sa_*

The presence of a catalytic triad involving a serine, a histidine and an aspartic acid was clearly suggested by previously described PHB depolymerases with known tertiary structure or catalytic mechanisms [34]. This fact is also supported by the clearly conserved amino acids found when performing a multiple sequence alignment (Figure 1). With the purpose of identifying the essential catalytic residues of PhaZ*_Sa_*, responsible for the hydrolysis of PHB, three specific amino acid-modifying reagents were used in this study (PMSF for serines, DEPC for histidines and glycinamide-EDC for carboxyl residues).

The residual PHB depolymerase activity after chemical modification of PhaZ*_Sa_* with different concentrations of modifying reagents can be observed in table 5; PMSF abolishes activity whereas DEPC and glycinamide-EDC exerted an important inhibition of the activity; these results support the presence of essential serine, histidine and carboxyl-residues for the catalytic activity of PhaZ*_Sa_*. Titration of sulfhydryl groups with Ellman’s reagent [26] revealed two free cysteine residues per enzyme molecule. Since PhaZ*_Sa_* has a total number of eight cysteine residues in its sequence, it means that two of these cysteines are in sulfhydryl form, and the other six are involved in the formation of three disulfide bonds, stabilizing the tertiary structure of the protein.

**Table 5:** Residual PHB depolymerase activity after chemical modification of PhaZ*_Sa_* by several amino acid-specific reagents.

Reagent	Concentration (mM)	Relative activity (%)
Control	–	100
PMSF	0.5	4
	1	0
DEPC	5	60
	20	10
EDC+Glycinamide	1	85
	5	67

In order to identify the serine, histidine and carboxyl-residues involved in the hydrolysis of PHB catalyzed by PhaZ*_Sa_*, site-directed mutagenesis of the enzyme was performed. Based on multiple sequence alignment, Ser^131^, Asp^209^ and His^269^ were strongly suggested as candidates for constituting the catalytic triad since they are strictly conserved. To demonstrate the importance of these putative catalytic amino acids, several mutant forms of the enzyme have been engineered by site-directed mutagenesis experiments. Five mutants of PhaZ*_Sa_* (namely S131A, S131C, D209N, H269E and H269Q) were expressed in *Rhodococcus* sp. T104; the correct secretion of these mutant enzymes to the extracellular medium was confirmed by SDS-PAGE (Figure S3–A). PHB depolymerase activity of cell-free culture broths was qualitatively checked by spot test activity assay. All mutant forms were correctly secreted by *Rhodococcus* sp. T104, and the PHB depolymerase activity was undetectable in every case (Figure S3-B). These results demonstrate the presence and identity of the catalytic triad in the active site of PhaZ*_Sa_*.

## Discussion

PHB depolymerases from many Gram-negative bacteria have been purified and are well-characterized in contrast to the limited knowledge of PHB depolymerases from Gram-positive bacteria. With this work, *Streptomyces ascomycinicus* has been proven to produce an extracellular PHB depolymerase (PhaZ*_Sa_*) specific for degradation of denatured and partially crystalline granules of PHB, present in the environment as a product of cell lysis of PHB-producer bacteria. Therefore, the role of this enzyme is not the mobilization of intracellular PHB, but the use of extracellular denatured PHB, in contrast to the hypothesis proposed by Wu et al. (5); in that work *fkbU* gene was proposed to encode a PHB depolymerase that could be responsible for maintaining the intracellular levels of butyryl-CoA during the stationary phase of growth, using the accumulated PHB as the carbon storage for this biosynthetic pathway. Since *fkbU* encodes an extracellular depolymerase, it cannot be directly related to the FK520 synthetic pathway, and despite its adjacent location, this gene should not be considered as a member of this cluster.

In addition, *fkbU* gene, encoding PhaZ*_Sa_*, has been cloned into pNV19 vector, expressed under control of the strong and constitutive ermE* promoter and secreted to extracellular medium by *Rhodococcus* sp. T104. The success of this expression system confirms the suitability of *Rhodococcus* sp. T104 for the heterologous expression of *Streptomyces* enzymes [9], and also demonstrates that this Gram-positive host recognizes genetic elements from *Streptomyces* genes, such as the ermE* promoter, and the consensus ribosome binding site, as well as secretory signal peptides. According to the predicted amino acid sequence of PhaZ*_Sa_* (accession number AAF86381.1) it presents an unusually long secretion putative signal peptide (58 residues), which predicted cleavage point was confirmed by N-terminal sequencing of the mature protein. However, *fkbU* could be expressed using an alternative translation start codon (GTG, residue number 33 in the predicted sequence) [12], preceded by a potential ribosome binding site (GGACG) located seven bp upstream, which would yield a 26 amino acids signal peptide, same length as reported for the PHB depolymerase from *S. exfoliatus* [9], [10]. This short signal peptide would fit the typical structure of secretory signal peptides from *Streptomyces* strains [40]. The analysis of PhaZ*_Sa_* secondary structure (Table 2) supports an alpha/beta hydrolase fold, present in many hydrolytic enzymes such as lipases. A catalytic domain type 1 according to the position of the putative oxyanion hole histidine (closer to the N-terminus than the catalytic amino acids) has been identified, as well as a substrate binding/recognizing domain type 1, with some clearly conserved amino acids (Figure 1) that might play an important role in the establishment of hydrophilic, hydrophobic or electrostatic interactions with the polymeric substrate [41], [42]. The catalytic triad (Ser^131^-Asp^209^-His^269^) which was previously postulated by multiple sequence alignment [34], is also present, as well as a lipase box pentapeptide (Gly-Leu-Ser-Ala-Gly), which includes the catalytic serine (Ser^131^). In this work, the identity of the catalytic triad has been confirmed by chemical modification and site-directed mutagenesis of specific amino acid residues.

**Figure 9:**
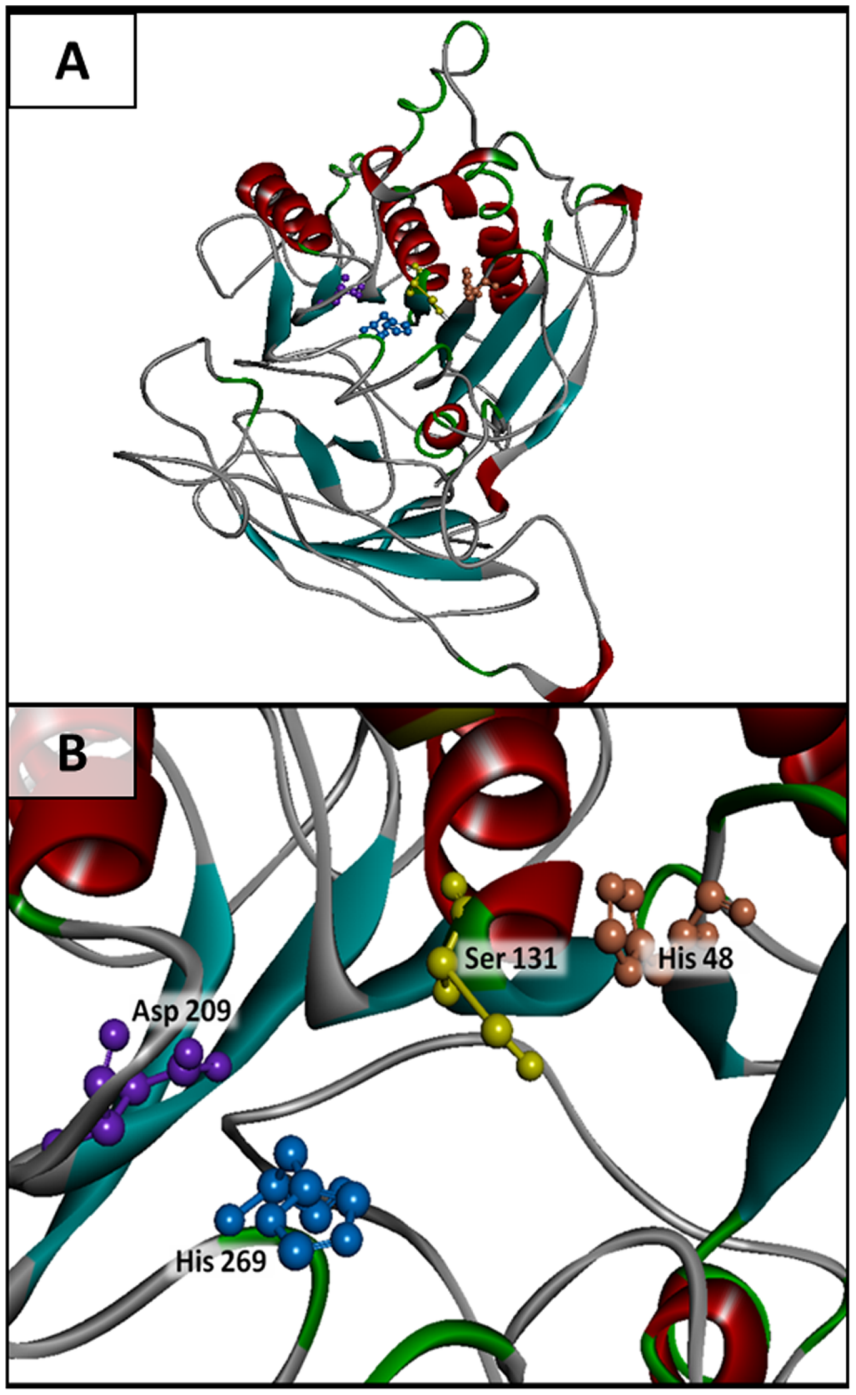
Three-dimensional structure model of PhaZ*_Sa_*. **A**) Complete model. This structure was modeled by the Phyre2 server (Protein Homology/analogY Recognition Engine V 2.0) [43] available at www.sbg.bio.ic.ac.uk/phyre2, which combines homology and *ab initio* modeling algorithms. These figures were rendered using Discovery Studio 3.1 software (Accelrys software Inc.) **B**) Detail of the active site, catalytic amino acids (Ser^131^-Asp^209^-His^269^) and oxyanion hole histidine (His^48^).

The homology modeling of the tertiary structure of PhaZ*_Sa_* using Phyre2 server [43] (Figure 9), suggested that this enzyme lacks a lid domain similarly to other described extracellular PHA depolymerases [44]. In this model the spatial arrangement of the catalytic amino acids can be observed, as well as the proximity of His^48^, the putative oxyanion hole histidine that stabilizes the tetrahedral transition state. The structure of the substrate binding domain has not been reliably established yet, due to the lack of homologous proteins with known three-dimensional folding, however, *ab initio* modeling of this domain provides an outline of its structure, which would comprise coiled regions and several β-sheets.

The extracellular expression of PhaZ*_Sa_* has facilitated its purification from the fermentation broth by only two hydrophobic interaction chromatography (HIC) steps. A good level of separation is achieved in the first HIC step (octyl sepharose), nevertheless a second HIC step (butyl HP sepharose) is required to reach a higher degree of purity and concentration, in order to characterize this enzyme.

Regarding the optimum reaction conditions, PhaZ*_Sa_* shows highest activity at 45°C, which is a very common optimum temperature among PHB depolymerases from soil microorganisms. However, the optimum pH of this enzyme is 6, this acidic pH is quite usual among fungal PHB depolymerases, but it is very uncommon in bacterial PHB depolymerases. Only two enzymes of this group, both from the Gram-negative *Ralstonia pickettii* have been reported to show acidic optimum pH values of 5.5 and 6 [45]. Hence, to the best of our knowledge PhaZ*_Sa_* is the first reported PHB depolymerase from a Gram-positive bacterium with an acidic pH optimum. This singular feature makes PhaZ*_Sa_* an interesting biocatalyst, suitable for PHB-derived residues degradation in acidic media.

Hydrolytic activity of PhaZ*_Sa_* is strongly enhanced (over 300%) in presence of low concentrations of divalent cations such as calcium, magnesium or zinc. However, PhaZ*_Sa_* remains slightly active even when it is thoroughly dialyzed in the presence of EDTA. These results indicate that PhaZ*_Sa_* shows a non-essential activation by divalent cations. Likewise, methyl-β-cyclodextrin (MβCD) exerts an important activating effect (up to 146%) at concentrations as low as 5 mM. This effect was previously described for PHB depolymerase from *Streptomyces exfoliatus* [9], but it remains unclear whether the effect of MβCD is produced directly on the enzyme, on the polymeric substrate (e.g. facilitating the access to PHB particles), or even associating with the reaction products, enhancing its solubility in the medium or shifting the equilibrium of the reaction towards the products.

Regarding kinetic parameters of PhaZ*_Sa_*, it is a difficult task to compare our data with those previously reported from other PHB depolymerases, since different measurement methods were employed in literature; moreover, the polymeric nature of the substrate is a problem when substrate concentration should be expressed in molarity terms. In this work, this problem was overcome by using the weight average molecular mass/M_W_ of the PHB, estimated by the manufacturer (437,000 g/mol), to calculate the moles of polymer degraded in activity assays. Using this approach, PhaZ*_Sa_* parameters (K_M_ 0.61 µM or 268 µg/ml and V_max_ 9,797 U/mg) can only be compared to those of PhaZ*Sex* from *S. exfoliatus* [9], which were obtained by the same procedure. In this sense, PhaZ*_Sa_* shows a 2-fold higher K_M_ value and 3.5-fold higher V_max_ value than PhaZ*Sex*, what means a better catalytic efficiency (1.75-fold higher). In general terms, when comparing K_M_ value of PhaZ*_Sa_* with those previously reported for other PHB depolymerases, several enzymes with higher affinity (lower K_M_) for PHB were found, such as PHB depolymerases from *Leptothrix* sp. HS [46], *Thermus thermophilus* HB8 [47] or *Paecilomyces lilacinus* D218 [48]. However PHB depolymerases with acidic pH optima (predominantly fungal) generally had a higher K_M_ value than PhaZ*_Sa_*, ranging from 0.69 to 14 mg/ml [49], [50], [51], [52]. This result supports PhaZ*_Sa_* as a suitable biocatalyst for PHB degradation in slightly acidic conditions.

Finally, PhaZ*_Sa_* has been successfully employed for degradation of PHB solvent cast films. Two PHBV copolymers with 5 or 12% hydroxyvalerate have been tested, as well as a blend of PHB and starch in 7∶3 (wt/wt) proportion. PHBV polymers containing both hydroxybutyrate and hydroxyvalerate monomers have many important advantages regarding their thermomechanical properties in comparison to the PHB homopolymer: they are more ductile, flexible, less crystalline and present better tensile strength and lower melting points [53], [54]. Likewise, blends of PHB with starch has been reported to confer improved mechanical properties as well, giving these films more tensile strength and increasing the extension needed to break them. Additionally, blending virgin PHB with starch would reduce considerably the high production costs of PHB without affecting its biodegradability [6]. In this work, PhaZ*_Sa_* has been proven to degrade all types of PHB films tested (Figure 7); the presence of starch or a moderate content of hydroxyvalerate in the film did not affect enzymatic depolymerization, although degradation of PHBV containing 12% hydroxyvalerate is relatively slower than the other tested bioplastics. During polyester degradation, (*R*)-3-hydroxybutyrate was released and quantified using a specific enzymatic assay, reaching approximately 40 mM concentration in the first 30 hours of incubation. Production of (*R*)-3-hydroxybutyrate by PhaZ*_Sa_* under mild conditions is an interesting feature, since this enantiomerically pure hydroxyalkanoic acid has a wide range of industrial and medical applications, serving as building block for synthesis of many fine chemicals or tailor-made plastics with controlled properties [55], [56].

To conclude, PhaZ*_Sa_* has very interesting properties for its industrial implementation, since it is an extracellular hydrolytic enzyme, stable up to 50°C and within a broad pH range (from 4 to 9), and is able to degrade different PHB copolymers. Moreover, PhaZ*_Sa_* can resist freezing and lyophilization with no need to add any protective additive, and its PHB depolymerase activity can be recovered even after denaturation with 6 M guanidinium chloride. These features make PhaZ*_Sa_* a promising candidate for the development of a robust biocatalyst able to degrade PHB-derived residues present in urban solid wastes.

## Supporting Information

Figure S1 **Production of recombinant PhaZ** ***_Sa_*** **in** ***Rhodococcus*** **T104.** **A)** SDS-PAGE analysis; Lanes 1–3: *Rhodococcus* T104 pNV19 (control strain). Lanes 5–7: *Rhodococcus* T104 pHPNV (*fkbU* clone). Lanes 1 and 5: culture broths, lanes 2 and 6: cell extracts, lanes 3 and 7: cellular debris, lane 4: Sigma wide range molecular weight standards. A protein band of about 50 kDa not present in the control strain is marked with an arrow in the culture broth of pHPNV clone. **B)** Spot test activity assay of different fractions of these strains. Wells are marked with their corresponding lane number in SDS-PAGE analysis from panel A.
(TIF)

Figure S2 **MALDI-TOF mass spectrum of pure recombinant PhaZ** ***_Sa_*** **expressed by** ***Rhodococcus*** **T104 pHPNV.**
(TIF)

Figure S3 **Site-directed mutagenesis of the catalytic triad residues of PhaZ** ***_Sa_*** **.** **A)** SDS-PAGE of the fermentation broths from the *Rhodococcus* T104 strains carrying the mutant pHPNV plasmids. Lane 1: S131A; lane 2: S131C; lane 3: D209N; lane 4: H269E; lane 5: H269Q; lane 6: pNV19 negative control; lane7; pHPNV positive control; lane 8: Bio-Rad broad range molecular weight standards. Band corresponding to PhaZ*_Sa_* or its mutant forms is marked with an arrow. **B)** Spot test PHB depolymerase activity assay of the fermentation broths containing the mutant forms of PhaZ*_Sa_*, the native PhaZ*_Sa_* and the negative control pNV19.
(TIF)

Table S1 **PCR primers used in this work for cloning of** ***fkbU*** **and site-directed mutagenesis of PhaZ** ***_Sa_*** **.**
(DOCX)

Table S2 **Purification table of PhaZ** ***_Sa_*** **.**
(DOCX)
