# Supplementary figures and images for: Novel Extracellular PHB Depolymerase from Streptomyces ascomycinicus: PHB Copolymers Degradation in Acidic Conditions
Source: PLoS One. 2013 Aug 12;8(8):e71699. doi: 10.1371/journal.pone.0071699 (PMC3741128; doi:10.1371/journal.pone.0071699)

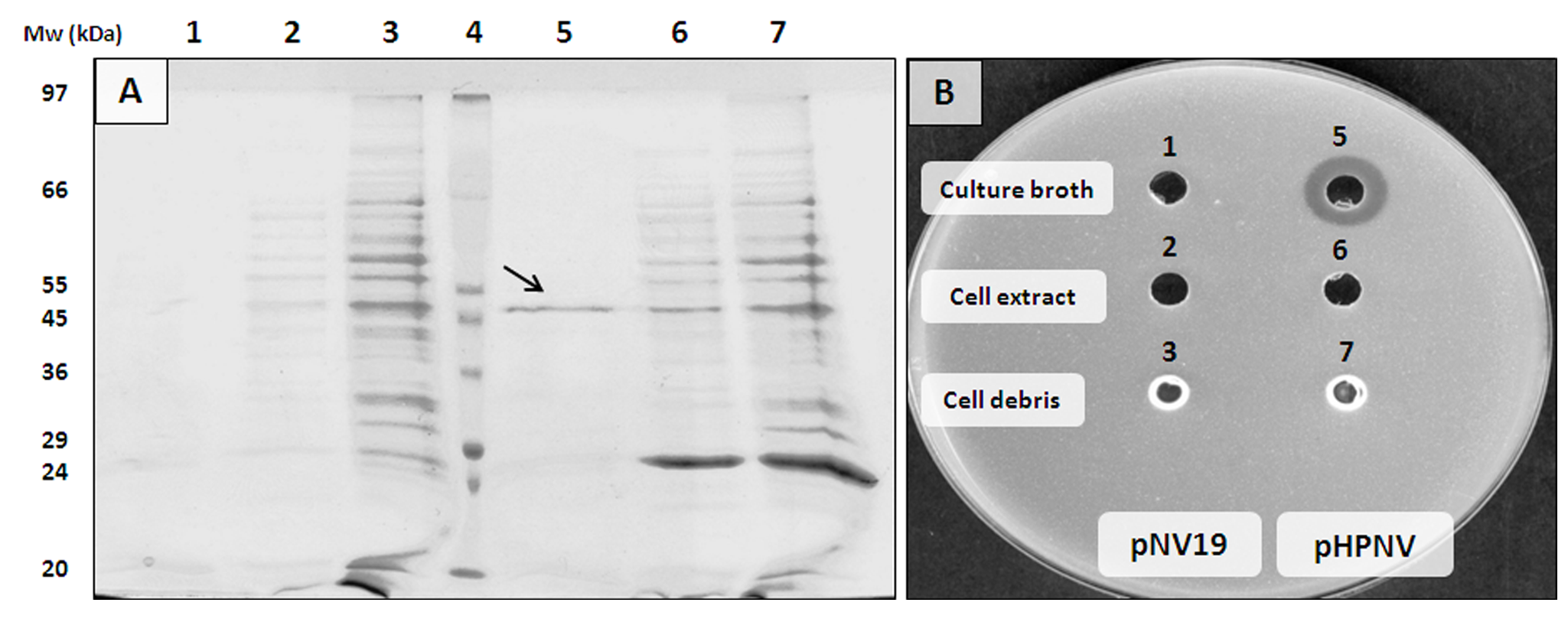

Supplement: Figure S1 — Production of recombinant PhaZ Sa in Rhodococcus T104. A) SDS-PAGE analysis; Lanes 1–3: Rhodococcus T104 pNV19 (control strain). Lanes 5–7: Rhodococcus T104 pHPNV (fkbU clone). Lanes 1 and 5: culture broths, lanes 2 and 6: cell extracts, lanes 3 and 7: cellular debris, lane 4: Sigma wide range molecular weight standards. A protein band of about 50 kDa not present in the control strain is marked with an arrow in the culture broth of pHPNV clone. B) Spot test activity assay of different fractions of these strains. Wells are marked with their corresponding lane number in SDS-PAGE analysis from panel A. (TIF) [file pone.0071699.s001.tif]

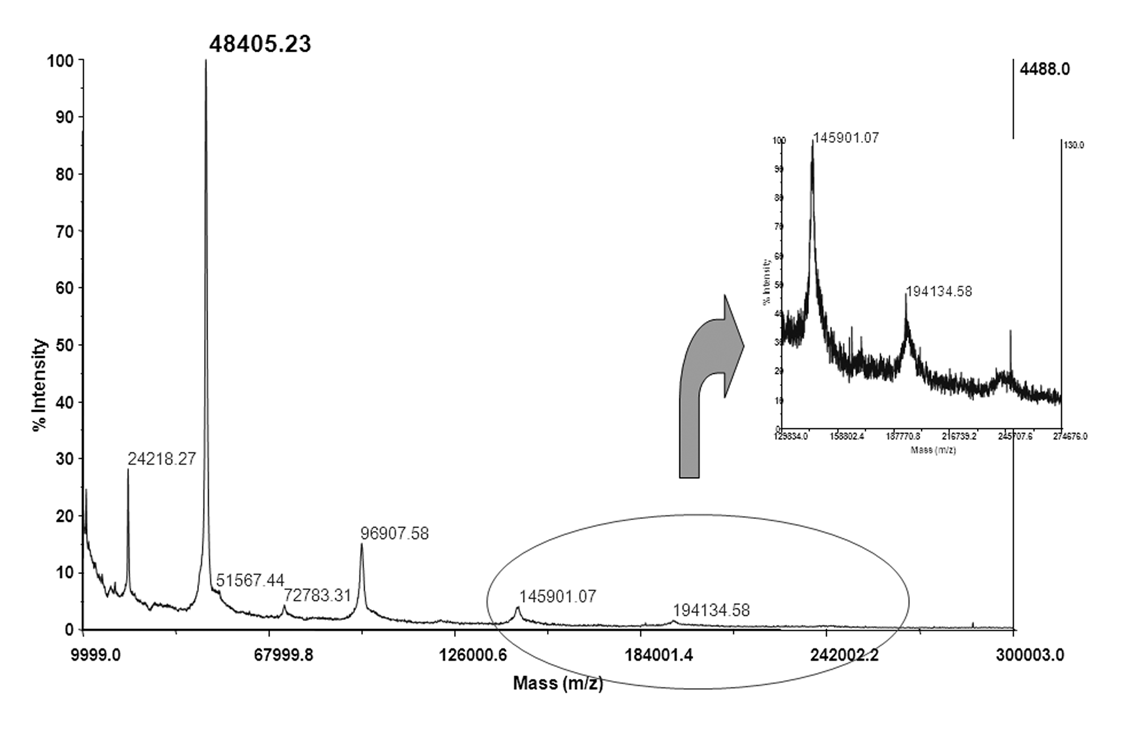

Supplement: Figure S2 — MALDI-TOF mass spectrum of pure recombinant PhaZ Sa expressed by Rhodococcus T104 pHPNV. (TIF) [file pone.0071699.s002.tif]

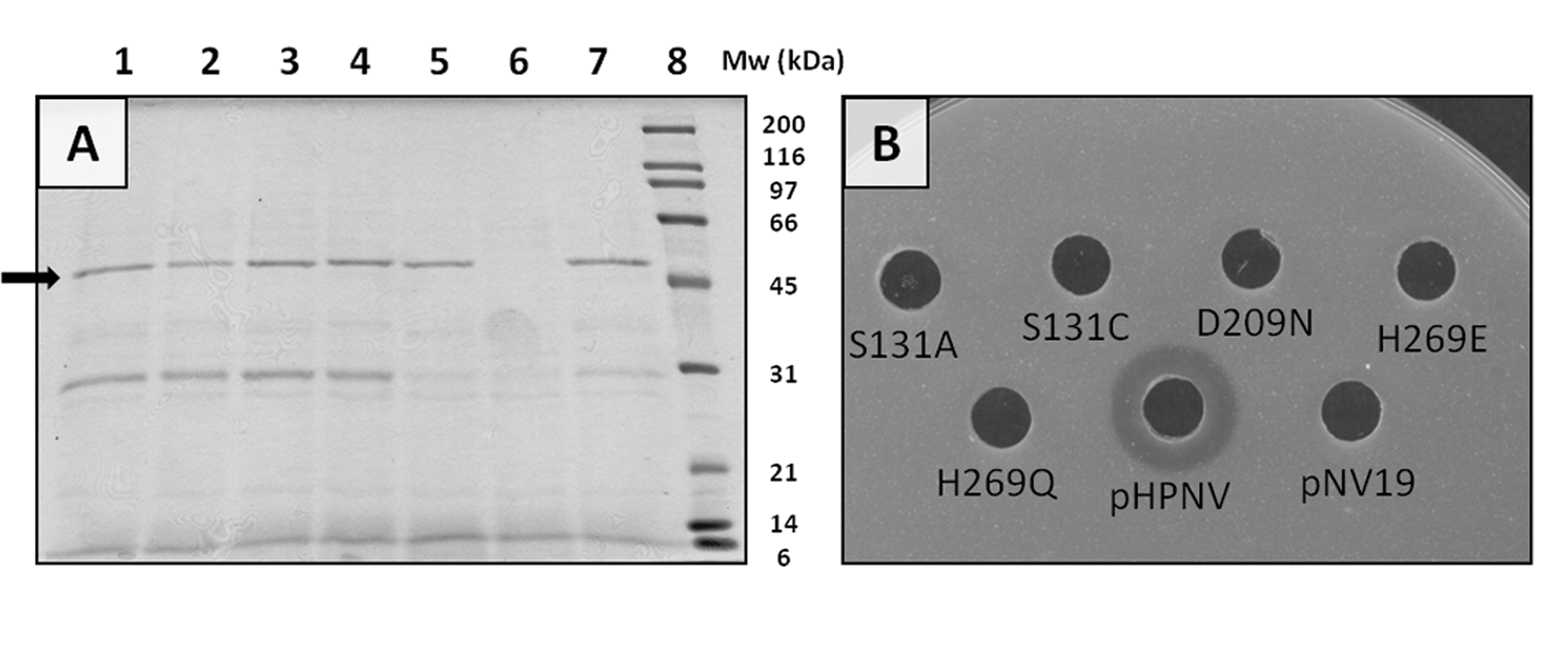

Supplement: Figure S3 — Site-directed mutagenesis of the catalytic triad residues of PhaZ Sa . A) SDS-PAGE of the fermentation broths from the Rhodococcus T104 strains carrying the mutant pHPNV plasmids. Lane 1: S131A; lane 2: S131C; lane 3: D209N; lane 4: H269E; lane 5: H269Q; lane 6: pNV19 negative control; lane7; pHPNV positive control; lane 8: Bio-Rad broad range molecular weight standards. Band corresponding to PhaZSa or its mutant forms is marked with an arrow. B) Spot test PHB depolymerase activity assay of the fermentation broths containing the mutant forms of PhaZSa, the native PhaZSa and the negative control pNV19. (TIF) [file pone.0071699.s003.tif]
