# Supplementary material for: Novel Extracellular PHB Depolymerase from Streptomyces ascomycinicus: PHB Copolymers Degradation in Acidic Conditions
Source: PLoS One. 2013 Aug 12;8(8):e71699. doi: 10.1371/journal.pone.0071699 (PMC3741128; doi:10.1371/journal.pone.0071699)
Supplement: Table S1 — PCR primers used in this work for cloning of fkbU and site-directed mutagenesis of PhaZ Sa . (DOCX) [file pone.0071699.s004.docx]

| TABLE S1: PCR primers used in this work for cloning of *fkbU* and site-directed mutagenesis of PhaZ*_Sa_* | | | | |
| --- | --- | --- | --- | --- |
| Primer name | **Sequence (5´→3´)*** | **Length (bp)** | **Tm (°C)** | **Restriction site** |
| HPET | catgccatggcaatgcagccgccgccgttccggggaatcctcacc | 45 | 96.7 | *Nco*I |
| HPEM | gctctagaggaggaacacccatgcagtcgccgccgttccgggg | 43 | 93.5 | *Xba*I |
| H3 | ggaattcctcagggacaggtgtcgttcgcgacggtgaagtagccg | 45 | 89.6 | *Eco*RI |
| S131A | caccgggctggccgccggcgg | 21 | 79.7 | - |
| S131A antisense | ccgccggcggccagcccggtg | 21 | 79.7 | - |
| S131C | acatcaccgggctgtgcgccggcg | 24 | 80.0 | - |
| S131C antisense | cgccggcgcacagcccggtgatgt | 24 | 80.0 | - |
| D209N | gcacggcgacaagaacaccaccgtcgc | 27 | 80.1 | - |
| D209N antisense | gcgacggtggtgttcttgtcgccgtgc | 27 | 80.1 | - |
| H269Q | cgggcatcgggcagggcacccc | 22 | 79.8 | - |
| H269Q antisense | ggggtgccctgcccgatgcccg | 22 | 79.8 | - |
| H269E | cgggcatcggggagggcaccccggt | 25 | 79.3 | - |
| H269E antisense | accggggtgccctccccgatgcccg | 25 | 79.3 | - |
| *Engineered restriction sites are shown in red. The mutated triplets of the site-directed mutagenesis primers are underlined. | | | | |
