# Supplementary material for: Novel Extracellular PHB Depolymerase from Streptomyces ascomycinicus: PHB Copolymers Degradation in Acidic Conditions
Source: PLoS One. 2013 Aug 12;8(8):e71699. doi: 10.1371/journal.pone.0071699 (PMC3741128; doi:10.1371/journal.pone.0071699)
Supplement: Table S2 — Purification table of PhaZ Sa . (DOCX) [file pone.0071699.s005.docx]

| TABLE S2: Purification table of PhaZ*_Sa_* | | | | | | | |
| --- | --- | --- | --- | --- | --- | --- | --- |
| Fraction | **Volume (ml)** | **Total Protein (mg)** | **Activity (U/ml)** | **Total Activity (U)** | **Specific Activity (U/mg)** | **Fold Purification** | **% Yield** |
| Culture Broth | 980 | 68,43 | 103 | 100450 | 1468 | 1,00 | 100 |
| Octyl Sepharose | 390 | 12,13 | 176 | 68510 | 5649 | 3,85 | 68 |
| Phenyl HP Sepharose | 220 | 6,10 | 172 | 37876 | 6212 | 4,23 | 38 |
